# The Tongue

**Published:** 1890-08

**Authors:** 


### THE TONGUE.

Taste is not equally distributed over the whole surface of the tongue. There are three distinct regions or tracts, each of which has to perform its own special office or function. The tip of the tongue is concerned mainly with pungent and acid tastes ; the middle portion is sensitive chiefly to sweets or bitters, while the back or lower portion confines itself entirely to the flavors of rich, fatty substances. This subdivision of faculties in the tongue makes each piece of food undergo three separate examinations, which must be successively passed before it is admitted into full participation in the human economy. The first examination gets rid of substances which would be actively and immediately destructive to the tissues of the mouth and body ; the second discriminates between poisonous and chemically harmless food, and the third merely decides the minor question whether the particular food is likely to prove wholesome or indigestible. The sense of taste proceeds, in fact, upon the principle of gradual selection and elimination ; it refuses first what is positively destructive, next, what is more remotely deleterious, and, finally, what is only undesirable or over luscious.

